# Case Report: Inflamed Jacobson nerve: an uncommon cause of persisting otalgia after an acute otitis media

**DOI:** 10.3389/fsurg.2024.1341118

**Published:** 2024-03-01

**Authors:** Aybige Camurdan, Alexander Kilgue, Lars Uwe Scholtz, Ingo Todt

**Affiliations:** Department of Otorhinolaryngology, Head and Neck Surgery, Medical Faculty OWL, Bielefeld University, Bielefeld, Germany

**Keywords:** otalgia, Jacobson's nerve, tympanic nerve, glossopharyngeal nerve, tympanoscopy

## Abstract

**Introduction:**

Otalgia can have multiple causes. Mostly otalgia is caused by a tubal dysfunction or an acute middle ear infection. This case describes a patient with an inflammation of the Jacobson's nerve causing severe persistent otalgia after an acute otitis media. The patients complaints completely disappeared after neurolysis of the Jacobson's nerve.

**Case presentation:**

We describe a case of a 21-year-old female caucasian patient with acute otitis media and persistent intractable otalgia. Infection was first successfully controlled by antibiotics. But the patient reported a persistent otalgia not responding to analgetics. We performed a CT scan, which exhibited a regular aerated middle ear finding, and a diagnostic tympanoscopy to examine the middle ear structures particularly the tympanic Jacobson's nerve as a possible cause for persistent pain. The following neurolysis of Jacobson's nerve under general anaesthesia led to a resolution of otalgia.

**Conclusion:**

An inflamed tympanic Jacobson's nerve is a rare observation and a persisting otalgia after an acute otitis media not responding to conservative treatment can be treated by a neurolysis.

## Introduction

Patients presenting with otalgia can be challenging. Anamnesis and examination of the ear usually help to distinguish many conditions. Otalgia is one of the most common symptoms of ear pathologies. Otalgia can be divided into two groups: *Primary* otalgia originates directly from ear pathologies such as otitis media or otitis externa for example.

*Secondary* otalgia originates from cranial nerves that innervate the ear (1).

The ear is innervated by the cranial nerve V (trigeminal), VII (facial), IX (glossopharyngeal), X (vagus) and C2 and C3 branches of the cervical plexus (2). Therefore, theoretically affections of all branches can cause otalgia. Clinically we observe this in cases of malignancies or temporomandibular joint disorders.

The tympanic branch of the glossopharyngeal nerve is called the Jacobson's nerve. This branch was first described by the Danish anatomist Ludwig Lewin Jacobson (1783–1843) in the 19th century (3). Jacobson also reported the canaliculus tympanicus, commonly known as Jacobson's canal, a passageway in the petrous bone that permits the tympanic branch of the glossopharyngeal nerve to pass through (3).

Literature about otalgia in terms of the role of the Jacobson's nerve is rare. There is one publication about Jacobson's nerve and otalgia describing traumatic neuroma of the tympanic Jacobson's nerve as a possible cause of otalgia (4). Additionally, a single-institution retrospective study with 13 ear patients has been conducted to analyze the efficacy of tympanic plexus neurectomy as a surgical intervention for patients experiencing refractory otalgia (7).

We report a patient case with otalgia due to inflammation of Jacobson's nerve caused by acute otitis media and direct recovery after neurolysis of Jacobson's nerve.

## Case presentation

**Figure 1:**
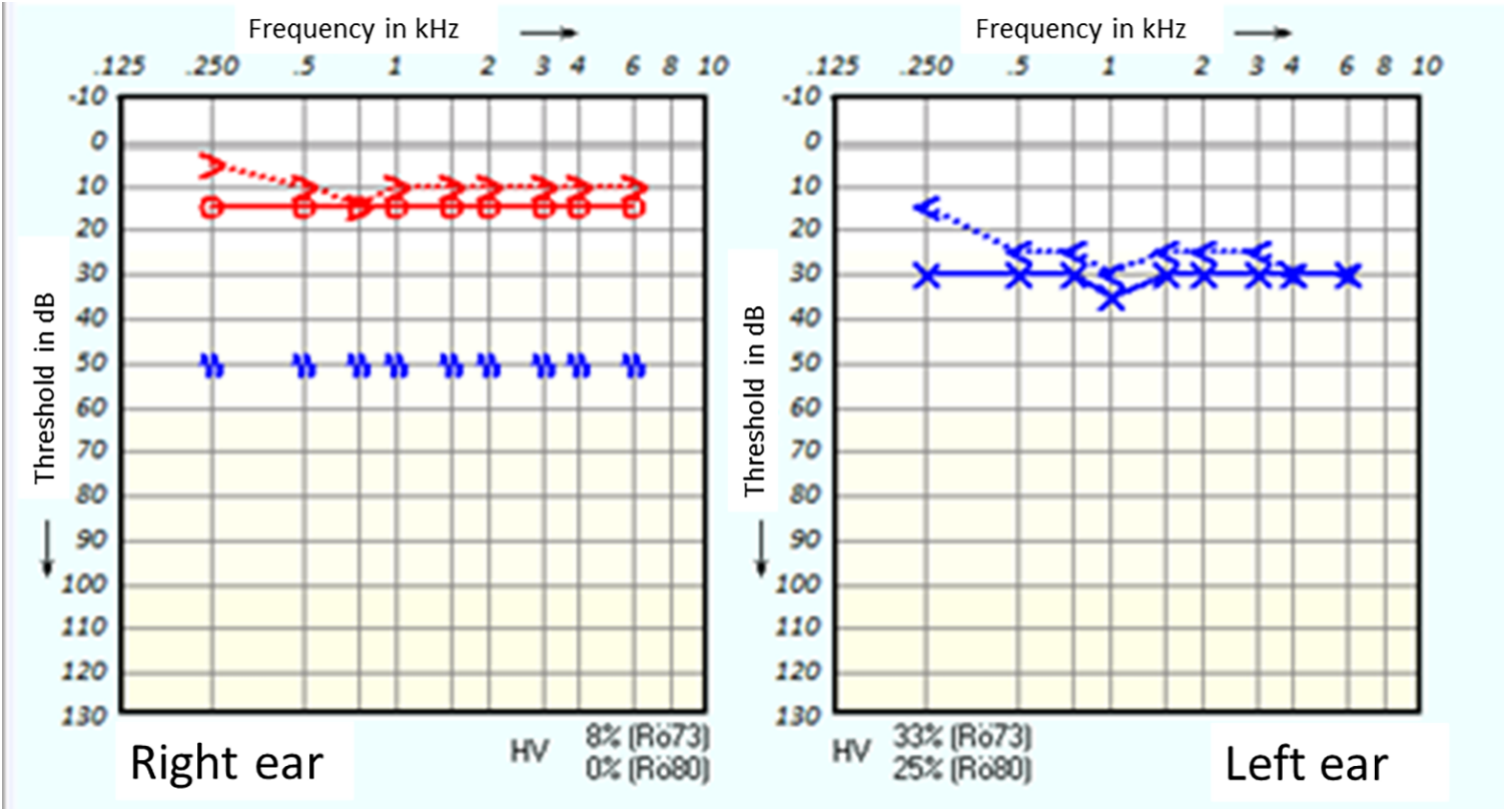
Pure-tone audiometry on admission day.

**Figure 2:**
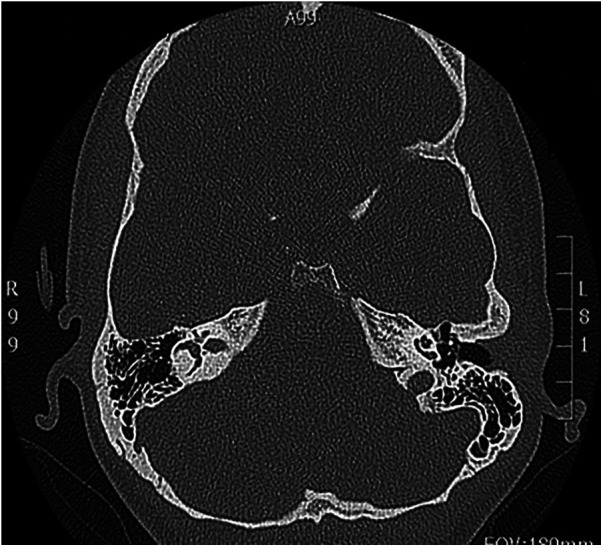
CT scan of temporal bones. Regular pneumatisation, regular configuration of ossicular chain.

We describe a case of a 21-year-old female caucasian patient with otitis media of the left ear, acute otalgia and mild hearing loss in pure-tone audiometry. The patient was treated with intravenous antibiotics and pain treatment according to WHO analgesic ladder. Intravenous cortisone therapy was given due to mild sensorineural hearing loss in pure-tone audiometry (Figure 1). Although the otoscopy image of otitis media and hearing threshold in PTA improved considerably, the patient was still complaining about persistent otalgia. A computer tomography of the temporal bones showed an unremarkable degree of pneumatisation and a normal configuration of the ossicular chain (Figure 2). The patient had no chronic disease, such as diabetes mellitus or any immunosuppressive disease.

**Figure 3:**
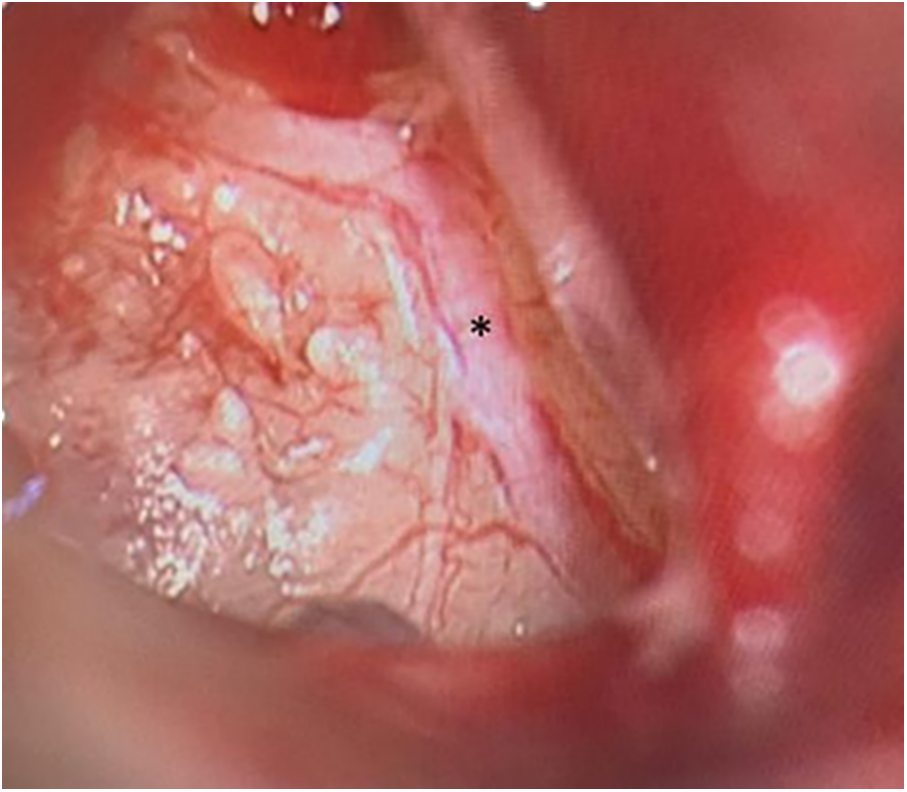
Tympanoscopy, intraoperative situs. Prominent Jacobson's nerve (*).

**Figure 4:**
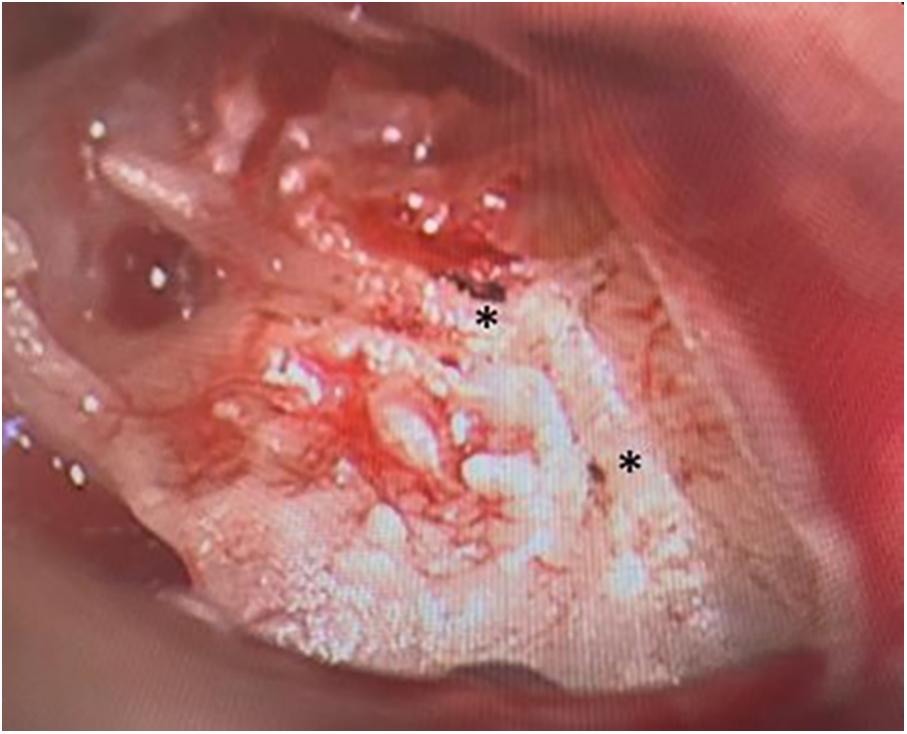
Tympanoscopy, intraoperative situs. Neurolysis of Jacobson's nerve in two areas (*).

For further diagnostic examination, we performed a diagnostic tympanoscopy of the left ear. A noticeable, very prominent tympanic Jacobson's nerve was detected (Figure 3). We performed a neurolysis of Jacobson's nerve by bipolar cauterisation to degenerate nerve fibres causing an interruption of pain signal transmissions (Figure 4). Immediate postoperative otalgia disappeared. After removal of the ear dressing, the hearing threshold in PTA remained the same as preoperatively. The patient did not report any complaints of otalgia in the postoperative clinical examination. In the following clinical examination, three weeks postoperatively the patient was free of pain symptoms. The sensorineural hearing loss in PTA improved in comparison to PTA on admission day.

## Discussion

The sensory innervation of the ear is complex. Sensation in the middle ear, external auditory canal, auricle and peri-auricular tissues is mediated by branches of the trigeminal nerve, facial nerve, glossopharyngeal nerve, vagus nerve, as well as branches of cervical nerves C1., C2. and C3. Otalgia can be caused by irritation of any one of these cranial nerves (5). The tympanic branch of the glossopharyngeal nerve is called the Jacobson's nerve (3). The function of Jacobson's nerve has not yet been fully understood. However a study has shown that Jacobson's nerve is important for the regulation of ear gas pressure in the middle ear (6). This case report highlights that inflammation of the Jacobson's nerve is an uncommon cause of persistent otalgia.

When no pathology is observed in the clinical and radiological examination of the ear and patients still remark persistent pain after proper treatment, examiners might occasionally tend to “psychosomatic pain” as a plausible cause for the discomfort. A further reason could be a virus-generated otitis media, which might even in this case explain the inner ear affection and the persisting pain. In this case, vesicles or skin affection was not observed, although even cases can occur without any visible effects. However, as shown in this case, the cause of an uncommon otalgia can be further investigated. Performing a diagnostic tympanoscopy to assess Jacobson's nerve under general anaesthesia can help to find a cause of persistent otalgia. A possible cause for an inflammation of the Jacobson nerve could be a neuroma, a scar formation, or a post-viral neuralgia. The persisting pain in this case remains unclear. In cases of ineffective conservative pain treatment, neurolysis can result in a reduction of transmitted neuronal pain signals and, therefore, a symptom-free situation. A placebo effect or a contribution of the change of the nerve supply by the tympanomeatal flap raise to the pain control seems unlikely but cannot be ruled out completely.

## Conclusion

Intractable persistent otalgia after acute otitis media may be caused by inflammation of Jacobson's nerve. Performing a diagnostic tympanoscopy following neurolysis of Jacobson's nerve can solve uncommonly persisting middle ear pain.

## Data Availability

The raw data supporting the conclusions of this article will be made available by the authors, without undue reservation.
